# The Causes and Circumstances of Drinking Water Incidents Impact Consumer Behaviour: Comparison of a Routine* versus* a Natural Disaster Incident

**DOI:** 10.3390/ijerph111111915

**Published:** 2014-11-18

**Authors:** Gabriella Rundblad, Olivia Knapton, Paul R. Hunter

**Affiliations:** 1 Department of Education and Professional Studies, King’s College London, Waterloo Road, London SE1 9NH, UK; E-Mail: olivia.knapton@kcl.ac.uk; 2 School of Medicine, Health Policy and Practice, University of East Anglia, Norwich NR4 7TJ, UK; E-Mail: paul.hunter@uea.ac.uk

**Keywords:** public health communication, compliance, routine incident, human error, natural disaster, drinking water

## Abstract

When public health is endangered, the general public can only protect themselves if timely messages are received and understood. Previous research has shown that the cause of threats to public health can affect risk perception and behaviours. This study compares compliance to public health advice and consumer behaviour during two “Boil Water” notices issued in the UK due to a routine incident* versus* a natural disaster incident. A postal questionnaire was sent to 1000 randomly selected households issued a routine “Boil Water” notice. Findings were then compared to a previous study that explored drinking water behaviour during a “Boil Water” notice issued after serious floods. Consumers affected by the routine incident showed a significant preference for official water company information, whereas consumers affected by the natural disaster preferred local information sources. Confusion over which notice was in place was found for both incidents. Non-compliance was significantly higher for the natural disaster (48.3%) than the routine incident (35.4%). For the routine incident, compliance with advice on drinking as well as preparing/cooking food and brushing teeth was positively associated with receiving advice from the local radio, while the opposite was true for those receiving advice from the water company/leaflet through the post; we suggest this may largely be due to confusion over needing boiled tap water for brushing teeth. No associations were found for demographic factors. We conclude that information dissemination plans should be tailored to the circumstances under which the advice is issued. Water companies should seek to educate the general public about water notices and which actions are safe and unsafe during which notice, as well as construct and disseminate clearer advice on brushing teeth and preparing/cooking food.

## 1. Background

Effective health communication is essential for disease prevention, health promotion and improved quality of life [1]. During public health emergencies, the general public can only protect themselves if messages from the response agencies are received and understood, preferably from a trustworthy source. When public health is threatened by contaminated mains water, water companies have the responsibility of issuing one of three water notices to their consumers: “Do Not Use”, “Do Not Drink” and “Boil Water”. In England and Wales, “Boil Water” notices are quite frequently issued, while “Do Not Drink” notices are very rare [2,3]. This paper explores the effects that the cause of the water incident and the circumstances under which the water notice was issued have on consumers’ behaviour. We will contrast results from a routine “Boil Water” notice study (*i.e.*, an incident that triggered a routine response) with results from a previous study of a natural disaster that involved a “Do Not Drink” notice immediately followed by a “Boil Water” notice [4].

Typically, risk communication is broken down into four elements: message, source, transmitter and receiver [5]. It is well known that a source’s perceived credibility will influence the public’s perceptions and behaviour, but it has proved challenging to identify which factors influence trust and credibility, especially at a local level. Interestingly, flavour and odour have been found to have the strongest relationship with more or less trust in the water companies [6]. An added difficulty is the potential impact that a transmitter can add to the mix, especially since government generated health advice is typically communicated through various media outlets, in particular, during emergencies [7].

Different information channel preferences have been found for natural* versus* routine incidents [8] and different channels may affect accuracy of risk knowledge [9]. During natural disasters, many agencies nowadays rely on transmission via television [10,11], radio [12], email or mobile phone [13]. Of crucial import is the timeliness of the advice, the loss of which will result in a lack of public comprehension of the advice, which in turn will increase confusion and anxiety, and reduce compliance levels [14]. Brodie and colleagues [15] found that about one-third did not get the message about the impending arrival of Hurricane Katrina at all, while a further one-third did not understand how to evacuate. Similarly, during Hurricane Rita, as few as 31% of people issued with a “Boil Water” notice were aware of it [16]. Some studies of transmitter effects during routine water incidents report that transmitter use has no effect on behaviour [17,18], while others report positive effects from interpersonal contacts [19] and negative effects from mass media [20]. Studies of transmitter effects during natural disaster water incidents report that consumers may depend upon word of mouth more than media sources [16].

Typically, non-compliance with ‘Boil Water’ advice ranges between 9% and 20% [18,21], whereas after Hurricane Rita, two-thirds did not boil their water for drinking [16]. If we include other ingestion actions, e.g., brushing teeth or preparing/cooking food, non-compliance increases dramatically to 57% and 77% for human error and natural disasters, respectively [16,21]. Recently, theories of public non-compliance have digressed from ideas of ‘irrational’ behaviour towards the idea that risky actions are choices resulting from individual and societal factors. At a personal level, these factors may include demographics [22], knowledge and experience of the situation [23] and general health beliefs [24]. Perceptions of the risks and recommended actions also influences compliance [25]; for example, high perceived risk has been found for involuntary rather than voluntary risks [26], unfamiliar rather than familiar risks [27] and risks that are not controllable by the individual [28]. In particular, technological mishaps are often perceived as high risk events [29]. Routine incidents that involve some degree of human error also tend to be regarded as actively imposed and unacceptable [30], whereas natural disasters are regarded as imposed without human agency and thus more acceptable.

Returning to the issue of trust, citizen advisory groups, health professionals, safety professionals, scientists and educators are consistently considered trustworthy or credible sources of information on environmental risk, except that those associated with an industry or believed to have a monetary interest are suspect sources [31,32]. Overall, it has been found that men have a greater level of trust in scientific authority, while women tend to exhibit higher levels of concern [33]. Older age is also consistently associated with greater trust in government, science and experts [34]. Climate change risk perception studies have found that highly educated people, whether measured in terms of general education or science education, tend to defer to scientific authority [35]. Due to the often inevitable interaction between demographics, as well as with other factors, such as trust and attitudes, it is often impossible to demonstrate the true impact of one single demographic variable upon water perceptions [36].

While consumers normally put great trust in information from physicians and other health professionals [19], in some studies, they are the source that consumers say they are least likely to consult about drinking water concerns [32]. Personal dissemination networks have been shown to be particularly vital for vulnerable sub-populations [11], and interpersonal information is often perceived as more credible and efficient than official information sources [19,37]. Thus, information source use and preferences might be a more relevant determinant in water safety communication, than demographics.

Of the four elements of risk communication, this paper primarily focuses on the effects of sources, transmitters and receivers upon consumer compliance to health advice. Through quantitative analysis, we show how different information sources and demographic characteristics affect consumer behaviour during the two water incidents. Undoubtedly, the message content also contributes to consumer understanding and behaviour. However, during the incidents in this study, the message content was a variable that we were unable to control for as both incidents involved a great number of sources and transmitters with a huge number of messages. In order to address issues such as how the linguistic framing of water advice might affect consumer compliance, we conducted a separate qualitative analysis of the water advice reported by the media during the natural disaster incident [38].

No previous studies have directly compared behaviour during routine water notices with behaviour during natural disaster water notices. We aim to show how the causes and circumstances of routine incidents* versus* natural disaster incidents result in differences in consumers’ compliance with advice, use of information sources, recall of advice received and their satisfaction with the information.

## 2. Methods

### 2.1. Two Incidents

At the Pitsford water treatment works in Northamptonshire in June 2008, a routine incident occurred after a rabbit entered the works [39]. Low levels of *cryptosporidium* oocysts were detected and a routine, precautionary “Boil Water” notice was issued to 258,000 people for ten days. In contrast, in summer 2007, the UK experienced its worst ever floods [40]. The Mythe water treatment works in Tewkesbury (Gloucestershire) was flooded, resulting in complete loss of water to 340,000 residents. When tap water was restored, consumers were issued a “Do Not Drink” notice for seven days, which was then replaced with a “Boil Water” notice for a further four days.

This section will outline the methods used to study the routine “Boil Water” incident at Pitsford. To ensure comparability, the same study design was employed here as in our previous study of the natural disaster incident at the Mythe water treatment work [4]. It should be noted that the distance between the two locations is 68 miles, but the households affected by the routine incident in 2008 had not been affected by the natural disaster incident in 2007.

### 2.2. Study Design and Sample Selection

A postal questionnaire study was sent to 1000 households affected by the routine incident in February 2009 (8 months after the incident). We obtained postcodes for affected areas from the Drinking Water Inspectorate. The Royal Mail Postcode Address File was then used to provide full addresses within these postcodes, from which 1000 were selected using a random number generator. Any business or school addresses were substituted for a further randomised selection of residential addresses.

Ethical approval was granted by the King’s College London Social Sciences, Humanities and Law Research Ethics Sub Committee.

### 2.3. Questionnaire Design

The postal questionnaire surveyed respondents’ uses of unboiled and boiled tap water, the advice that they remember receiving and the information sources that they consulted. Mainly close-ended questions were employed, which were a combination of yes/no questions, ranking questions, and “tick only one” and “tick as many as apply” multiple choice questions. The questionnaire was piloted twice on undergraduate students from King’s College London (N = 50), and minor revisions were made to wordings. The final questionnaire was sent out with a detailed project description and a stamped, addressed return envelope. A reminder was sent four weeks later to those who had not yet replied.

### 2.4. Coding

For ranking questions, where participants ticked rather than ranked options, a single tick was coded as rank one, whereas multiple ticks were given the same rank (e.g., three ticks were ranked as 2). Where participants first ranked options but then ticked one further option, the tick was coded as their lowest rank. On “tick only one” questions, multiple ticks were coded as inconclusive, with the exception of the water advice recollection question where multiple ticks were coded as “believed more than one advice was in place” so that uncertainty could be accounted for. For information source questions where respondents ticked “other’ or “website” but then provided additional information, answers were re-coded so that e.g., “television” includes listening, phoning and visiting websites of television channels/programmes whereas “website” includes internet-only sources. Open-ended questions were quantified where possible; e.g., home ownership was translated into the binary categories “yes, home owner” and “no, not home owner”. For all questions, non-responses were coded as missing data and inconclusive replies were largely excluded from analysis.

### 2.5. Analyses and Hypotheses

Data were entered into Microsoft Access 2007 and then cross-checked against the original responses. For statistical analysis, data were transferred into SPSS version 16. Once the data had been transferred into SPSS, they were validated a second time. As some respondents did not fully answer some questions, the sample size varies between questions.

As the routine incident only affected the drinking water, that is, other resources such as traffic, communications and electricity were unaffected, we hypothesised *apriori* that non-compliance with water advice would be higher for the natural disaster event compared to the routine incident. In addition, we predicted that demographic factors (such as age, gender, home ownership and employment, which were coded and explored identically for both studies), drinking water preferences, and use of information sources could have had an effect on participants’ perceptions and behaviours; however, as no formal hypotheses were defined *apriori* for the impact of demographics, drinking water preferences and use of information sources, statistical outcomes for these variables should be interpreted solely as indicators of the potential strength of association. Quantitative analysis is mainly descriptive. Inferential analysis was carried out using Chi-Square, Mann-Whitney, ANOVA, and Linear Regression. For all analyses with multiple predictor variables, only those variables that were significant at the *p* < 0.2 level in single predictor models were included in the multiple predictor models. The least significant variable was then removed from the model until all predictor variables were significant at the *p* < 0.2 level. The value of the model in predicting each dependent variable was then derived from the tests of between subjects’ effects in the corrected model. Throughout, the level of significance was set at 5% and only responses with at least 10 responses were included as dependent variables.

## 3. Results

This section outlines the key findings from the study of the routine incident, and compares results to the findings from the “Boil Water” notice stage of the natural disaster study [4].

### 3.1. Response Rate and Demographics

In total, 173 completed questionnaires were returned from consumers who receive mains water from Pitsford water treatment works. Eleven respondents stated that they had not been issued with the “Boil Water” notice. These were excluded from analysis, yielding a sample size of 162 participants. This routine incident response rate of 17.3% is comparable to the 19.5% rate for the natural disaster.

We compared the demographic characteristics of the routine respondents with those for the disaster participants, to check for sampling bias [41]. Overall, there were no significant differences between the two samples (Table 1). With regards to ethnicity, it should be noted that both samples were overwhelmingly white (97.5% and 98.7%, respectively).

**Table 1:** Demographics of routine incident and natural disaster respondents.

Demographics	Routine	Disaster	*p*
**Gender**			0.493 **^a^**
male	58	62	
female	102	93	
**Age**			0.057 **^b^**
20 or under	2	0	
21–30	16	10	
31–40	27	21	
41–50	35	27	
51–60	23	35	
61–70	33	36	
over 70	25	29	
**Home ownership**			0.169 **^a^**
yes, home owner	130	138	
no, not home owner	29	20	
**Occupation**			0.745 **^a^**
yes, in paid employment	85	85	
no, not in paid employment	69	64	

**^a^** Chi-Square; **^b^** Mann-Whitney U.

### 3.2. Information Sources

As Table 2 depicts, routine incident consumers primarily consulted the water company (76.5%), while natural disaster consumers did not show a clear preference [4]. Use of information sources was consistently higher for the routine incident, with the exception of local radio. Similarly, the average number of information sources utilised during the routine incident was significantly higher at 2.833 (Range = 0–8) compared to 1.791 (Range = 0–4) for the natural disaster event (U = 5498.5; *p* = 2.1 × 10^−9^ (2-tailed)).

**Table 2:** Use of information sources by routine incident and natural disaster respondents.

Information Source	Routine N = 162		Disaster N = 115		*p *^a^
	n	%	n	%	
family/friend/neighbour	59	36.4	12	10.4	1.1 × 10^−6^
local newspaper	67	41.4	32	27.8	0.021
water company/leaflet through the post	124	76.5	66	57.4	0.001
TV	89	54.9	19	16.5	1.0 × 10^−1^°
local radio	78	48.1	64	55.7	0.218

**^a^** Chi-Square

Participants were also asked to rank the information sources in order of how useful they had found each source for the entire incident. For the routine incident, the water company was ranked highest (41.2%, N = 119), whereas for the natural disaster, local radio was ranked highest (53.4%) [4].

### 3.3. Advice

The vast majority of routine incident consumers reported receiving water advice (96.9%, n = 157/162). However, there was a lot of confusion regarding which notice was in place (Table 3). Quite strikingly, more than 41% of consumers believed that there were two simultaneous notices, whereas 47% recalled a “Boil Water” notice. Similar confusion was found for the natural disaster incident, with only 71.4% of disaster consumers recalling receiving advice being given and 26.7% recalling that advice as “Boil Water” [4].

**Table 3:** Advice recollection.

Advice Recollection	(N = 157)	
	n	%
there was one advice: do not use	9	5.7%
there was one advice: do not drink	6	3.8%
there was one advice: boil	74	47.1%
there was one advice: safe	3	1.9%
there was more than one type of advice	65	41.4%

We queried how clear the tap water advice was and how well-informed routine incident consumers had felt: 65.0% (n = 104/160) thought the advice was “clear” or “very clear” and 90.1% (n = 145/161) felt “informed” or “very informed”. In comparison with the natural disaster (Table 4), more routine incident consumers felt the advice was “clear” or “very clear”, and their feeling of being informed was more frequently described as “very informed”.

For the routine incident, advice from the water company/leaflet through the post was positively associated with clear advice and feeling informed; no other information source displayed any association (Table 5). In the natural disaster incident, use of local newspapers was positively associated with clear advice, and use of the water company was associated with feeling informed [4].

**Table 4:** Comparison of clarity of advice and feeling informed between routine incident and natural disaster respondents.

Appreciation of Advice		Routine		Disaster		*p *^a^
		n	%	n	%	
clarity of advice **^b^**	very unclear	4	2.5	10	7.1	0.010
	unclear	9	5.6	8	5.7	
	understandable	43	26.9	41	29.1	
	clear	54	33.8	59	41.8	
	very clear	50	31.3	23	16.3	
feeling informed **^c^**	very uninformed	5	3.1	12	8.5	3.8 × 10^−4^
	uninformed	11	6.8	11	7.7	
	informed	90	55.9	97	68.3	
	very informed	55	34.2	22	15.5	

**^a^** Chi-squared for trend; **^b^** Routine N = 160; Disaster N = 141; **^c^** Routine N = 161; Disaster N = 142.

**Table 5:** Final parameter estimates of ANOVAs of predictors of clarity of advice and feeling informed.

Dependent Variable	Predictor Variables	B	LCI	UCI	p
clarity of advice	water company/leaflet through the post	0.657	0.297	1.017	4.2 × 10^−4^
feeling informed	water company/leaflet through the post	0.344	0.090	0.599	0.008

### 3.4. Compliance and Water Behaviour

Routine incident respondents were asked to specify their use of unboiled and boiled tap water (Table 6). Some consumers drank unboiled tap water and used it to make babies’ bottles. It should also be noted that some consumers were overcautious in their behaviour by using boiled tap water or avoiding unboiled tap water for flushing the toilet, washing hands and showering.

**Table 6:** Use of unboiled and boiled tap water by routine residents.

Activity	Use of Unboiled Water (N = 161)		Use of Boiled Water (N = 161)	
	n	%	n	%
flush toilet	144	89.4	2	1.2
shower/bathe	142	88.2	5	3.1
wash hands	117	72.7	24	14.9
prepare/cook food with **^1^**	40	24.8	100	62.1
brush teeth **^1^**	34	21.1	90	55.9
drink cold **^1^**	8	5.0	81	50.3
prepare babies' bottles **^1^**	2	1.2	6	3.7

**^1^** Action not safe with unboiled water if a “Boil Water” notice is in place.

We measured compliance both for drinking behaviour and ingestion behaviour. Respondents who did not drink tap water straight from the tap were considered compliant with drinking advice. Ingestion behaviour also included tap water used for preparing/cooking food and brushing teeth. Comparison of rates for the routine incident and the natural disaster showed that both drinking water compliance (95.0% *vs.* 70.7%) and overall ingestion compliance (64.6% *vs.* 51.7%) was significantly better for the routine incident, as confirmed by Chi-Square (Table 7). The high level of compliance with drinking water advice for the routine incident could not be traced to any specific factors.

**Table 7:** Comparison of compliance between routine incident and natural disaster respondents.

Compliance	Routine N = 161		Disaster N = 116		*p *^a^
	n	%	n	%	
Drinking water compliance	153	95.0	82	70.7	2.5 × 10^−8^
Ingestion compliance	104	64.6	60	51.7	0.032

**^a^** Chi-Square.

We recoded ingestion compliance on a scale from 0 to 3 (0 = no compliance, 3 = full compliance). For the routine incident, advice from the local radio was positively associated with compliance with ingestion advice, whereas advice from the water company/leaflet through the post was associated with less compliance (Table 8).

**Table 8:** Linear Regression with final predictor variables of compliance with advice on ingestion.

Dependent Variable	Predictor Variables	B	LCI	UCI	*p*
Ingestion compliance	water company/leaflet through the post	−0.324	−0.613	−0.035	0.028
	local radio	0.305	0.060	0.550	0.015
	general drinking preference	0.277	−0.025	0.578	0.072

**Figure 1:**
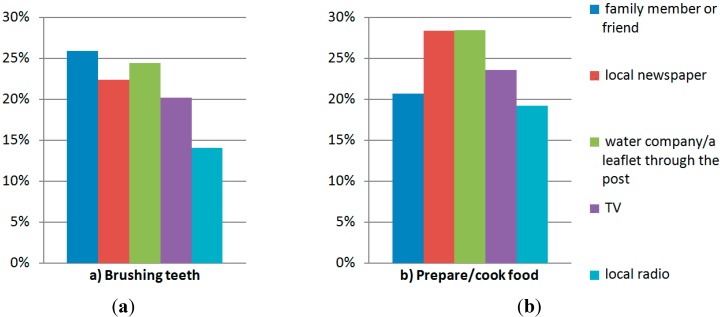
Percentage of information source users who did not comply with the advice (**a**) not to brush teeth and (**b**) not to use for preparation/cooking of food with unboiled tap water.

As Figure 1 illustrates, consumers who received advice from the local radio seem more likely to comply with the advice not to use unboiled tap water for brushing teeth and food preparation/cooking, than those that received advice from the water company/leaflet through the post.

## 4. Discussion and Conclusions

Following the 2007 floods in the UK, the Pitt Review predicted a marked increase in extreme weather and with it natural disaster incidents [40]; these predictions were realised in February 2014 when the same areas as in 2007 were again flooded, as well as many neighbouring areas. This paper has sought to contrast perceptions and behaviour in the British general public for two incidents that resulted in a “Boil Water” notice, where one was triggered by a routine water incident (*i.e.*, the incident triggered a routine response) and the second by the 2007 floods.

### 4.1. Key Findings

Information source use differed between the two incidents, and the use of information sources was significantly higher for the routine incident. Here, the water company was the most accessed information source and it was also ranked as the most useful. For the natural disaster, on the other hand, the water company and the local radio were used in almost equal measure, but the local radio was ranked as the most useful [4]. We found a higher degree of satisfaction with water advice for the routine incident. Those who used the water company leaflet and/or contacted the water company felt more informed and felt the advice was clearer.

Previous routine incident studies have found that between 9% and 20% of respondents drink unboiled tap water despite the “Boil Water” notice [17,18,21]. For overall ingestion behaviour, non-compliance for such incidents have been recorded as high as 64% (or 81% if washing plates is included as overall ingestion) [18]. Non-compliance for the routine incident investigated here was 5% (drinking) and 35% (ingestion), thus somewhat lower than normal. In comparison, non-compliance for the natural disaster was consistently higher (29.3% for drinking and 48.3% for ingestion).

Both the greater satisfaction with water advice and the notably lower degree of non-compliance for the routine event may be attributable to the fact that Anglian Water’s communication campaign included vans with hailers and information points in the streets. In contrast, the floods of 2007 often impaired many communication methods. The natural disaster incident may have triggered a higher than normal need for geographic-specific information [4], but it is also very likely that the high use of local media was reinforced by the fact that there were over 25 agencies involved [42], resulting in the water company not being as prominent an authority source [38].

The discrepancy in satisfaction and non-compliance highlights the need for water companies to establish themselves as the primary information source for all water incidents, so that in case of a future incident, whether it be a natural disaster one or a routine one, media and other agencies refer to their advice. It should also be pointed out that for the routine event it was the local radio that was associated with higher ingestion compliance with advice, whereas the water company/leaflet had the opposite effect. Thus, the role of local media as an additional and effective way to disseminate emergency health advice is clear and it is therefore vital that efforts to keep them continuously up-dated during an event are prioritised. It is also essential that all sources of information are instructed to clarify that the need to boil the water extends to all ingestion activities.

### 4.2. Language and Cognition

Even though it is the legal responsibility of the water company to provide temporary water supplies during a water supply failure, Knapton and Rundblad [38] found that during the Mythe natural disaster event the water company was continually hidden in media reports through linguistic techniques such as implication and ellipsis. The water company’s lack of prominence in the media reports may have hindered the public’s comprehension of the water company’s responsibilities, which could have led to increased confusion over to whom to turn for water advice. Additionally, the lack of visibility of the water company within the reports may indicate a general gap in the public’s knowledge about water companies’ roles and duties during water incidents. Improving public understanding of water companies’ duties could lead to increases in the public’s trust in the water company and the public’s compliance to water advice.

The finding that 41.4% of routine incident consumers believed that there was more than one simultaneous notice echoes the early stages of the natural disaster when a “Do Not Drink” notice was in place (35.2%). These parallel results from the routine incident reinforce Rundblad* et al.*’s [4] conclusion that the public are not aware of the exclusivity of water notices, but rather employ a binary categorisation of water as “safe” or “unsafe”. For both incidents, we also found that approximately 30% of consumers were, on average, behaving overcautiously; e.g., two elderly, female, retired consumers used boiled tap water to flush the toilet. Similarly inappropriate protection efforts have been found in climate risk studies, where older adults applied sunscreen but did not prevent dehydration as they confused heat wave risk and UV radiation risk [43]. Recall of simultaneous notices and uncertainty over which actions are safe, therefore, are not simply a result of the general confusion of a natural disaster, but pervade through society even under routine circumstances. Due to the range of risky and overcautious behaviours displayed here, future studies need to address the public’s knowledge of notices.

Interestingly, a recent study on perceptions about water contaminants found that in an incident-free context, boiling and chilling tap water to drink was highly unfavoured [33]. In addition, when asked about changes to water behaviour, consumers were very unlikely to use boiling as a treatment at home. Similarly, questions asking which types of water were treated and tested enough, did not reveal a higher belief in the quality of boiled tap water (29%–42%) compared to unboiled tap water (38%–48%). Bottled water quality, on the other hand, was felt to exceed both of these (65%–75%). These findings could be interpreted as in stark contrast to Rundblad* et al.*’s [4] suggestion of a strong folk-belief in boiling since during the “Do Not Drink” notice part of the Mythe natural disaster event, many consumers did not comply accurately, but instead treated their tap water by boiling it before consuming it. These contrasting results warrant further exploration in future studies.

Additionally, Knapton and Rundblad [38] found that the general public’s agency throughout the incident was often omitted in the media reports through various linguistic techniques. In combination with this backgrounding of the general public, the water advice was constructed using linguistic techniques that do not promote a sense of obligation to comply. For example, epistemic modal verbs of choice (rather than deontic modals of necessity) and ambiguous action words such as *use* (rather than specific actions such as *drink*) were common. As a result, the reporting of the water advice could actually have lessened the public’s motivation to comply with that advice and to take personal responsibility for their own safe water behaviours. There are, to our knowledge, no studies of linguistic techniques in media reports during routine water events.

### 4.3. Strengths and Weaknesses

In the present study of the routine incident, we found no impact of demographic factors on compliance with water advice. It should be noted that whilst there were also no associations between demographics and behaviour for the “Boil Water” notice period of the natural disaster incident, our earlier study did find that whether a person was in paid employment or not was significantly associated with use of unboiled water for ingestion purposes during the “Do Not Drink” notice period [4]. These findings add to the discussions on the influence of demographic factors [44,45] by suggesting that social factors play a more important role during natural disasters than routine incidents. It is essential that public health education reaches all income quartiles and all ages. In addition, because demographics also affect hygiene (which does not represent a risk under a “Do Not Drink” or “Boil Water” notice), social backgrounds could identify those consumers who are more likely to behave overcautiously and thus potentially experience greater levels of anxiety.

Assessing current knowledge of public compliance to water notices is complicated by the various methodologies of previous studies. Whilst some have measured use of unboiled water [18,21], others have measured use of boiled water [16], thereby seriously limiting comparability. This study measured use of both unboiled and boiled water, allowing greater depth of analysis and comparison. By employing the same study design as a recent natural disaster study, combined with no significant differences between the demographic compositions of the two samples, this is the first study to compare information use, advice recollection, compliance and water behaviours between routine water notices and natural disaster water notices.

Response rates for both the present study and our previous natural disaster study were just below 20%. In the past, surveys of compliance with water notices that were sent out shortly after the incident have yielded response rates of around 65% [17]. However, it is an increasingly common problem for unsolicited postal surveys to receive low response rates [46]. For example, a recently published study also focussing on the 2007 floods yielded a response rate of only 12% [47], and Risebro and colleagues’ [48] study of contaminated small drinking water supplies yielded 14% and 11% for Norfolk/Suffolk and Herefordshire, respectively. As these low response rates may have reduced the statistical power in our study, we urge that caution should be exerted when generalising these results.

Previous studies have shown that the greater the time gap between an event and data collection regarding that event, the greater the negative effect on recall accuracy [49]. Largely, correct recall depends on the willingness of the participants to recall the event, which in turn is dependent on the importance of the event to the participant [50]. In her study of the September 11 events, Pezdek [51] found that event memory was most accurate for participants who were physically closer to the events, presumably because these were more personally involved and distressed by the events, which in turn triggered more narrative rehearsal. For the present study, the time elapsed between incident and questionnaire was for the routine event 8 months and for the natural disaster 18 months. In the latter case, the time gap may have led to inaccurate recall of events and behaviours. However, during focus groups held in the areas affected by the floods of 2007 [4], we found that the disaster was of such an unprecedented scale that we find it unlikely for participants to have forgotten significant details. Even so, the time delay does place some restrictions on our results and conclusions.
